# PCR and dissection as tools to monitor filarial infection of *Aedes polynesiensis *mosquitoes in French Polynesia

**DOI:** 10.1186/1475-2883-5-2

**Published:** 2006-02-24

**Authors:** Catherine Plichart, Yves Sechan, Neil Davies, Anne-Marie Legrand

**Affiliations:** 1 Institut Louis Malardé, P0 Box 30, Papeete, 98713 Tahiti, French Polynesia; 2 Centre IRD (Institut de Recherche pour le Développement), PO Box 529, Papeete, 98713 Tahiti, French Polynesia; 3 Richard B. Gump South Pacific Research Station, University of California Berkeley, PO Box 244, 98728 Moorea, French Polynesia

## Abstract

**Background:**

Entomological methods may provide important tools for monitoring the transmission of filariasis in French Polynesia. In order to standardize our PCR method and refine our protocol to assess filarial infection levels in mosquitoes, we compared dissection of the vector, *Aedes polynesiensis*, with the poolscreening polymerase chain reaction (PS-PCR) assay.

**Methods:**

(1) Mosquitoes were collected in human landing catches in five areas in Moorea island, French Polynesia. (2) A fraction of the captured mosquitoes was dissected for *Wuchereria bancrofti *larvae. (3) Laboratory-reared mosquitoes (uninfected as well as experimentally infected ones) were repeatedly tested to optimize a PS-PCR protocol (DNA extracts from 1–50 pooled mosquitoes were tested with an internal standardized system and primers specific for the Ssp1 repeat sequence. PCR products were analysed by gel electrophoresis). (4) Another fraction of the captured mosquitoes was assayed by PS-PCR according the optimized protocol.

**Results:**

The prevalence of field-mosquito infection with *W. bancrofti *ranged from 1 to 8 % by dissection (L1–L3) and point estimates of infection prevalence, as assayed by PS-PCR, ranged from 0.4 to 3.7 %. There was a moderately strong correlation between larval infection rates as determined by dissection and PCR.

**Discussion:**

Our results suggest that the PS-PCR assay is specific and highly sensitive for detecting parasite DNA. We obtained similar although not identical results with dissections of mosquitoes. PS-PCR appears to be adequate for testing large numbers of mosquitoes in the context of filariasis elimination programs. The role and advantages of using entomologic methods to monitor filariasis programs are discussed.

## Background

Nearly 10 % of the population in French Polynesia is still infected with *Wuchereria bancrofti *filariae with the Society and Marquisas archipelagos being the most heavily infected. Monitoring the efficacy of filariasis elimination programs depends on careful evaluation of infection levels in the human and vector populations following the introduction of control programs.

French Polynesia has a long history of filariasis control. The most recent effort to achieve filariasis elimination in French Polynesia is the Pacific programme for the Elimination of Lymphatic Filariasis (PacELF). PacELF was launched in 2000 and is based on annual mass drug administration (MDA) for a minimum of five years of entire populations with a combination of diethylcarbamazine at a dosage of 6 mg/kg of body weight and albendazole (400 mg/person). In French Polynesia, the MDA is carried out begining every April. The impact of the community treatment program in sentinel sites (Maupiti, Raiatea and Tahuata) will be reported elsewhere. Here, we focus on the assessment of infections in the mosquito-vectors. For ethical reasons, monitoring infections in mosquitoes offers some advantages over repeated blood examinations of the human population. Mosquito dissection has been the gold standard for measuring infection rates and densities in the vector. However, it becomes increasingly costly and laborious to carry out adequate numbers of dissections in areas where the infection prevalence drops below 1%. The ability of PCR techniques to detect one microfilaria in pools of up to 50 mosquitoes suggests that current PCR methods could facilitate the testing of adequate numbers of mosquitoes to enable changes in transmission to be measured.

In this study, we describe a standardized PCR protocol to assess filarial infection levels in mosquitoes. To validate our standardized procedure, we assessed filarial infection rates in field-captured *Ae. polynesiensis *and compared PS-PCR with dissection. The study area was Moorea island in the Society archipelago because its location close to Tahiti facilitated repeated field mosquito collections. Even though we had no recent data on the prevalence of LF in the human population of this island, data of previous investigations [1,2] (41% microfilaremia prevalence in 1950 before any MDA, 0.4% in 1983 after near 30 years continuous DEC active prophylaxis and 7% in 1993 after a ten year-stop of any MDA) let us suppose a "moderate" permanent transmission level.

## Methods

### Collection sites

The main vector for *W. bancrofti *in French Polynesia is the day-biting mosquito *Ae. polynesiensis *[3] with transmission occuring year-round. Mosquitoes were collected at five locations in the Communes of Teavaro and Afareaitu on the east-south-east area of Moorea, Society Archipelago : Afareaitu, Haumi, Maatea, Teavaro and Vaiare.

### Mosquito collection method

As Ae. polynesiensis cannot be captured in adequate numbers in any commercially available traps, it was necessary to capture the mosquitoes in human landing catches [3]. Mosquitoes were collected by aspiration as they landed for a blood meal. Collections were made within 15 meters of a house. In general, collection locations were separated by at least 150 meters. During the collection period, the human acting as the "bait" bared his legs and sat quietly, while the catcher circled slowly catching the mosquitoes as they landed. All mosquitoes collected from a single station were placed in a labeled cotton stoppered test tube at 5–8°C in an ice-box until transported to the laboratory.

### Sampling strategy for field mosquitoes

*Ae. polynesiensis *populations were sampled by collecting as many female mosquitoes within 10 minutes periods at each collection sites. Captured individuals were representative of the population in terms of density and infection status. A conscious effort was made to select the shadiest and most protected location within the collection site to ensure the greatest likelihood of catching mosquitoes. Two full 10 minutes collections were undertaken at every collection "station" irregardless of the mosquito biting density. The number of collection stations per collection site ranged between 9 to 19 depending on the geographical area of the site in order to obtain a representative sample of that study area.

### Laboratory-reared mosquitoes

The laboratory colony of *Ae. polynesiensis *was reared in 30-cm3 cages kept in an environmental chamber at 27–29°C, 80–90% relative humidity on a 16:8-hour light:dark cycle [4]. Larvae for the colony were collected from natural breeding sites (crab burrows, small ponds and puddles) and placed in a rearing tray filled with 1 L of distilled water. Larvae were fed powdered cat pellets. Adult mosquitoes were allowed access to a cotton wick soaked in 10 % sucrose solution. Eggs to maintain the colony were obtained by feeding mosquitoes on laboratory rats.

### Mosquito dissection

Mosquitoes were usually dissected within six hours after they were collected; in a few instances they were held overnight at 4°C and dissected the following day. Just before starting the dissection and for one tube at a time, mosquitoes were anesthetized by chilling for a few minutes. Then, they were placed on a petri dish and the wings and legs removed using two pairs of jewelerss' forceps under low power magnification. The individual mosquitoes were then placed on a microscope slide and with dissecting needles divided into head, thorax and abdomen, placing each portion of the body in a separate drop of saline solution on the same slide. The abdomen was dissected first and ovaries graded for parity [5]. Nulliparous females were immediately registered as uninfected without further dissection. For parous females, the three body segments were teased apart and examined for larval stage worms or microfilaria. The location and number of the parasites were noted. Mosquitoes carrying microfilaria, L1, L2 or L3 larvae were defined as infected.

### Experimental infection of laboratory-reared mosquitoes

Artificial blood-feeding of reared female mosquitoes was performed using the Parafilm membrane feeding method according to Failloux et al. and Rutledge et al. [4,6]. Mosquitoes were orally infected via a blood meal taken through a Parafilm membrane placed over the opening of a feeding reservoir. Infectious blood was prepared using an appropriate amount of microfilariae recovered by the membrane filtration technique [7] from the venous blood of an infected person. Microfilariae were then resuspended in uninfected blood (added with ATP, 2.6 mg/mL) from a donor at a density of 1,200–1,500 microfilariae per mL (determined by a triplicate counting of 2 μL samples). Feeding reservoirs were filled with 3.5 mL of infected blood and allowed to warm to 37°C. Feeders were then placed on the top of plastic cups covered by a nylon net. Cups containing 50 6-day old females were starved for 24 hours prior to membrane feeding. Mosquitoes were allowed to feed for 1 h. Engorged females (125/400) were kept ; 20 were immediately dissected and the others frozen at -20°C until used as positive controls for DNA extraction (mean number of microfilariae per mosquito was found to be 1.2 ± 1.4; n = 20).

### Extraction of DNA

DNA extractions were performed according to the method described by Nicolas et al. [8] with some modifications (method A) or using the Qiagen DNAeasy Tissue Kit (Qiagen, Hilden, Germany; Cat. No. 69504 with a procedure specially optimized for mosquito tissues (method B). Pools of mosquitoes were dried for 3 h at 90°C, prior to being carefully crashed with a sterile pestle tissue grinder (Fisher Scientific) in a 1.5 mL eppendorf tube.

#### Method A

The crushed material was cleaned twice with 1 mL of washing buffer (0.1 M NaCl, 30 mM Tris-HCl, pH 8, 30 mM EDTA, 10 mM 2β-mercaptoethanol and 0.5 % Nonidet P40^®^), centrifuged (Eppendorf Microfuge^®^, at 15,800 g for 1 min) and the supernatant discarded. DNA was extracted by incubating the pellet in 200 μL of lysis buffer (0.1 M NaOH and 0.2% sodium dodecylsulphate) for 1 h at 37°C, and then neutralized with 10 μL of 2 N HCl. After a centrifugation step (3 min at 15,800 g), supernatant was transferred into a new tube. For DNA binding on silica beads, the extract was then mixed thoroughly with 1 mL of a 5 M guanidine hydrothiocyanate solution added with 1.2% Triton X100^® ^in TE buffer (20 mM EDTA, 50 mM Tris-HCl, pH 6.4) and 40 μL of a suspension of silica beads (Sigma-Aldrich, St Louis, MO, USA; Ref. S-5631, 40 mg/sample; before use, silica was washed twice in distilled water, then suspended in HCl solution, pH 2 and kept at 4°C). After 10 minutes incubation at room temperature to allow DNA adsorption and a centrifugation step (1 min at 15,800 g), the silica beads were washed twice with 1 mL of a 5 M guanidine hydrothiocyanate solution in 50 mM Tris-HCl buffer (pH 6.4) and twice with 1 mL of ethanol 70%. The silica beads were then carefully dried at 56°C for 10 min, resuspended in 100 μL of TE buffer (1 mM EDTA, 10 mM Tris-HCl, pH 8) and incubated at 56°C for 10 min to elute the DNA. After a centrifugation step (3 min at 15,800 g), the purified DNA sample in the supernatant was kept at -20°C until used.

#### Method B

was performed according to manufacturer's recommendations with the following modifications: The crushed material was homogenized with 180 μL PBS and the tube spun briefly. Lysis buffer (AL, 200 μl) was added and two incubation steps with proteinase k were performed. The first incubation was for 10 min at 70°C with proteinase k (20 μL) and a second incubation was for 1 h at 56°C with another 20 μL of proteinase k. After a centrifugation step (5 min at 15,800 g), the supernatant was transferred into a new tube to get rid of large cuticle debris, aqueous ethanol (98%, 200 μL) was added before applying the whole sample (including any precipitate) to the spin silica column for DNA binding. The column was washed twice with 500 μl buffer AW1 and once with 500 μl buffer AW2 and then carefully dried by spinning twice (3 min at 15,800 g). Finally, DNA was eluted from the column in a labeled tube adding elution buffer (AE, 120 μl, twice). The purified DNA sample obtained was kept at -20°C until used.

### DNA extraction controls

Two negative DNA extraction controls from a pool of reared mosquitoes were included with all runs. Three positive controls were also included with each PCR run. Positive controls consisted of pools of reared mosquitoes spiked with one experimentally infected mosquito (mean number of microfilariae per infected mosquito was 1.2 ± 1.4, n = 20, as determined by microscopy) or with microfilariae (mean number of microfilariae per 2 μL sample counted under microscope: 3 ± 2, n = 6).

### PCR amplification

The PCR assays were conducted using NV-1 and NV-2 oligonucleotides specific for the S*sp 1 *repeat as previously described by Zhong et al., Chanteau et al. and Nicolas and Plichart [10-12]. Amplification was performed with a Mastercycler gradient PCR thermocycler (Eppendorf AG, Hamburg, Germany) using Hot Start Taq (Qiagen, Hilden, Germany) and 1/20^e^, 1/40^e ^or 1/240^e ^of DNA sample in a reaction volume of 25 μL. The PCR temperature program was as follows: 15 min at 94°C, 5 min at 54°, then 30s at 72°C, 20s at 94°C, 30s at 54°C for 35 cycles, and finally 5 min at 72°C. Reaction mix included 1 unit of Taq polymerase, 5 pmoles of each primer, NV1 and NV2, 5 nmoles of each dideoxynucleotide and 1.5 μL of 25 mM MgCl_2 _in 1× Qiagen PCR buffer. The efficiency of PCR amplification may vary from sample to sample, depending on the quality of DNA preparations. Some PCR inhibitors from mosquitoes may not be removed completely leading to false negative results. To control for this variation, in a second tube an internal standard (pWb11) was added to each PCR reaction for co-amplification along with the target *W. bancrofti *DNA using the same pair of primers. A negative control for the PCR assay, using sterile distilled water instead of DNA extract in the reaction mix was included with all runs as well as a PCR positive control (*Ssp1 *sequence cloned in a plasmid pTZ18, this recombinant plasmid is designated pWb01). Analysis of PCR products was performed via gel electrophoresis. Ten μL of the PCR product were loaded onto a 1.75% agarose gel with ethidium bromide (0.5 μg/mL) for UV visualization of the amplicon bands (191 bp for the Ssp1 repeat and 225 bp for the pWb11 internal control).

### Statistical analysis

Prevalence rates from dissection were expressed as a percentage of infected mosquitoes and calculated with 95% confidence intervals. PCR point estimates were computed and compared using Poolscreen 2.0 generously provided by Dr. Tom Unnasch and Charles Katholi (The University of Alabama, Birmingham, USA) [13]. The Pearson correlation coefficient was used to estimate the association of dissection based prevalence rates with PCR based rates.

## Results

### Collection and dissection data

*Ae. polynesiensis *female mosquitoes were collected in different sites of the study area during three time periods. The goal of field-collection 1 (October 2003) was to gather preliminary data about capture rates and approximate larval prevalence in dissected mosquitoes. Thereafter, field-collection 2 (November 2003) was managed in order to collect sufficient *Ae. polynesiensis *to be analysed in 2 batches for comparison between individual dissections and the PS-PCR method. The third field-collection (April 2004) assessed prevalence levels under different seasonal conditions from those of collection 2. Field data are presented in table 1.

**Table 1:** Field-collection data and infection rates in dissected (parous and nulliparous) mosquitoes

**Collection** ** sites**	**Collection** ** dates**	**Number** ** stations**	**% station** ** with Ap**	**Capture** rate *	**Number** ** captured Ap**	**Number** ** dissected Ap**	**% parous in** ** dissected Ap**	**Infection** rate **
**Afareaitu**	Oct. 03	11	60%	0.2	60	60	77%	7 % [1–13]
	Nov. 03	19	100%	1.6	890	216	94%	8 % [5–12]
	Apr.04	19	80%	0.4	337	0	nd	nd
**Haumi**	Nov. 03	10	100%	2.4	724	179	97%	2 % [0–4]
**Maatea**	Oct. 03	10	100%	0.7	212	212	43%	3 % [1–6]
	Nov. 03	15	100%	4.2	1899	478	97%	1 % [0–2]
	Apr.04	15	70%	0.8	478	0	nd	nd
**Teavaro**	Nov. 03	9	100%	0.5	272	67	94%	3% [0–7]
**Vaiare**	Oct. 03	8	50%	0.1	22	22	82%	4,5% [0–13]
	Nov. 03	13	100%	0.9	342	81	99%	6 % [1–11]
	Apr.04	13	60%	0.3	166	0	nd	nd

* capture rate is obtained by dividing the number of minutes spent in catching effort into the number of mosquitoes caught during that time

** percentage of mosquitoes carrying *W. bancrofti *larvae (microfilaria, L1, L2 or L3) [95% confidence interval]

Mosquito collections at different periods differed in their capture rates. Clearly, these parameters (obtained by dividing the number of minutes spent in catching effort into the number of mosquitoes caught during that time) varied significatively in Afareaitu, Maatea and Vaiare by collection period. Capture rates in April 2004 were 20% to 33% of the capture rates observed in November 2003 for exactly the same collection "stations".

During field-collection 2 in November 2003, 25% of the mosquitoes captured (in each collection station of each collection site) were kept for dissection. Parity rates were usually between 80 and 100%. Infection rates as determined by dissection and counting of those infected with *W. bancrofti *L1, L2, or L3 larvae, showed some variation from one collection site to another, with infection levels ranging between 1 to 8 % (table 1).

Among the 1,021 dissected mosquitoes during field-collection 2 in November 2003, 34 were found to be infected with *W. bancrofti *(table 2). They harboured mostly L1 and L3 larvae with mean numbers of larvae for each larval stage ranging between 1 and 4 per infected mosquito.

**Table 2:** Wuchereria bancrofti larvae in infected mosquitoes collected during field-collection 2 in November 2003

**Collection sites**	Infected Ap *	L1 larvae **	L2 larvae **	L3 larvae **
**Afareaitu**	18/216 8 % [5–12]	49 2.6 ± 5.8	6 0.3 ± 0.9	36 1.9 ± 2.9
**Haumi**	3/179 2 % [0–4]	2 0.7 ± 1.1	0 0	13 4.3 ± 6.7
**Maatea**	6/478 1 % [0–2]	2 0.3 ± 0.8	0 0	15 2.5 ± 2.5
**Teavaro**	2/67 3% [0–7]	0 0	4 2 ± 2.8	15 2.5 ± 2.4
**Vaiare**	5/81 6 % [1–11]	13 2.6 ± 2.9	1 0.2 ± 0.4	6 1.2 ± 2.7
**Total area**	**34/1021 ** **3% [2–4]**	**66 ** **1.9 ± -4.5**	**11 ** **0.3 ± 1**	**85 ** **2.4 ± 3.8**

* : number of infected mosquitoes over number of dissected mosquitoes and percent infection rates.

** : total number of larvae found and mean number of larvae per mosquito

### Optimizing DNA extraction and PCR procedure with laboratory-reared mosquitoes

We evaluated different parameters to optimize the DNA extraction procedure using the Qiagen kit (B method) to obtain results similar in sensitivity and specificity to the reference method (A method). First, special care was taken for fine grinding of the mosquito tissues. Second, in order to obtain a complete lysis of the cell membranes for optimal release of the DNA, incubation with proteinase K was performed twice. Furthermore, attention was given to the washing steps of the DNA extract bound on the silica column in order to obtain optimal cleaning and to eliminate inhibitors (compounds liable to further inhibit the PCR).

Table 3 shows the results obtained with pools of laboratory-reared mosquitoes spiked with one experimentally infected mosquito extracted using respectively method A and method B. In both cases, we observed that eleven of the 12 positive controls yielded two amplified DNA bands (191 bp and 225 bp). One case failed to yield the 191 bp band although the 225 bp internal standard was efficiently amplified. These control extracts correspond to mosquitoes who failed to ingest microfilariae during the experimentally infected blood meal. With the B method, we observed that positive and negative controls failed to yield amplified DNA bands (191 bp band as well 225 bp band) when 1/20^e ^or 1/40e of the DNA extracts were used for PCR. Such results signal the presence of inhibitors at a dose that blocks the Taq polymerase activity. Decreasing the DNA sample concentration to 1/240e or 1/1200^e ^leads to DNA amplification, indicating that inhibitory compounds are not in sufficient concentration to be active. Positive controls prepared with microfilariae spiked in pools of laboratory-reared mosquitoes and extracted using the B method gave reliable positive results without inhibition (table 4) when the fraction used for PCR was small (1/240e i.e 1 μL of the template). A trial to reduce the amount of Taq polymerase used gave false negative results (data not shown) so we retained 1 U as the optimum amount of Taq polymerase.

**Table 3:** Results for *Wuchereria bancrofti *DNA extraction in pools of laboratory-reared uninfected mosquitoes added (positive) or not (negative) with one experimentally infected mosquito.

**Pool size**	**Method A (Reference)**	**DNA sample fraction**	**191 bp band**	**225 bp band**	Résults *
**1**	positive controls	1/20e	+ (n = 3/3)	+ (n = 3/3)	+ (n = 3/3)
	negative control	1/20e	- (n = 1)	+ (n = 1)	- (n = 1)
**20**	positive controls	1/20e	**+ (n = 2/3)**	+ (n = 3/3)	**+ (n = 2/3)**
	negative control	1/20e	- (n = 1)	+ (n = 1)	- (n = 1)
**30**	positive controls	1/20e	+ (n = 3/3)	+ (n = 3/3)	+ (n = 3/3)
	negative control	1/20e	- (n = 1)	+ (n = 1)	- (n = 1)
**50**	positive controls	1/20e	+ (n = 3/3)	+ (n = 3/3)	+ (n = 3/3)
	negative control	1/20e	- (n = 1)	+ (n = 1)	- (n = 1)

**Pool size**	**Method B (Qiagen kit)**	**DNA sample fraction**	**191 bp band**	**225 bp band**	**Résults**

**1**	positive controls	1/20e	+ (n = 3/3)	+ (n = 3/3)	+ (n = 3/3)
		1/40e	+ (n = 3/3)	+ (n = 3/3)	+ (n = 3/3)
		1/240e	+ (n = 3/3)	+ (n = 3/3)	+ (n = 3/3)
	negative control	1/20e	- (n = 1)	+ (n = 1)	- (n = 1)
		1/40e	- (n = 1)	+ (n = 1)	- (n = 1)
		1/240e	- (n = 1)	+ (n = 1)	- (n = 1)
**20**	positive controls	1/20e	- (n = 3/3)	- (n = 3/3)	**inh (n = 3/3)**
		1/40e	- (n = 3/3)	- (n = 3/3)	**inh (n = 3/3)**
		1/240e	+ (n = 2/3)	+ (n = 2/3)	**inh (n = 1/3)**
		1/1200^e^	+ (n = 3/3)	+ (n = 3/3)	+ (n = 3/3)
	negative control	1/20e	- (n = 1)	- (n = 1)	**inh (n = 1)**
		1/40e	- (n = 1)	- (n = 1)	**inh (n = 1)**
		1/240e	- (n = 1)	- (n = 1)	**inh (n = 1)**
		1/1200^e^	- (n = 1)	+ (n = 1)	- (n = 1)
**30**	positive controls	1/20e	- (n = 3/3)	- (n = 3/3)	**inh (n = 3/3)**
		1/40e	- (n = 3/3)	- (n = 3/3)	**inh (n = 3/3)**
		1/240e	**+ (n = 2/3)**	+ (n = 3/3)	**+ (n = 2/3)**
	negative control	1/20e	- (n = 1)	- (n = 1)	**inh (n = 1)**
		1/40e	- (n = 1)	- (n = 1)	**inh (n = 1)**
		1/240e	- (n = 1)	+ (n = 1)	- (n = 1)
**50**	positive controls	1/20e	- (n = 3/3)	- (n = 3/3)	**inh (n = 3/3)**
		1/40e	- (n = 3/3)	- (n = 3/3)	**inh (n = 3/3)**
		1/240e	+ (n = 3/3)	+ (n = 3/3)	+ (n = 3/3)
	negative control	1/20e	- (n = 1)	- (n = 1)	**inh (n = 1)**
		1/40e	- (n = 1)	- (n = 1)	**inh (n = 1)**
		1/240e	- (n = 1)	+ (n = 1)	- (n = 1)

**Table 4:** PCR amplification of *Wuchereria bancrofti *DNA in pools of laboratory-reared uninfected mosquitoes spiked with microfilariae

**Pool size**	**Control testing**	**191 bp band**	**225 bp band**	**Résults**
**0**	Mf spiked controls	+ (n = 3/3)	+ (n = 3/3)	+ (n = 3/3)
	Negative control	- (n = 1)	+ (n = 1)	- (n = 1)
**20**	Mf spiked controls	+ (n = 3/3)	+ (n = 3/3)	+ (n = 3/3)
	Negative control	- (n = 1)	+ (n = 1)	- (n = 1)
**30**	Mf spiked controls	+ (n = 3/3)	+ (n = 3/3)	+ (n = 3/3)
	Negative control	- (n = 1)	+ (n = 1)	- (n = 1)
**50**	Mf spiked controls	+ (n = 3/3)	+ (n = 3/3)	+ (n = 3/3)
	Negative control	- (n = 1)	+ (n = 1)	- (n = 1)

* DNA extracted according the B method; sample fraction used 1/240^e^

### Applying the optimized protocol to field-collected mosquitoes

The optimized protocol for DNA extraction using the Qiagen kit and PCR amplification of a 1/240^e ^fraction of the DNA extract was applied to pools of 20 mosquitoes collected in Moorea. The infection of female A*e. polynesiensis *by *W. bancrofti *was assessed both by individual dissection and PS-PCR in mosquitoes from field-collection 2 (see table 5 and figure 2). There was a moderately strong and significant correlation between larval infection rates as determined by dissection and PCR (r = 0.68, p < 0.05). Mosquitoes collected in April 2004 showed PCR estimated larval prevalences similar to the rates observed in November 2003 (table 6), although the number of mosquitoes collected in the total area was significantly smaller.

**Table 5:** *Wuchereria bancrofti *larval (L1–L3) prevalence in dissected ^1 ^and PCR-poolscreened ^2 ^mosquitoes collected in november 2003

**Collection sites**	**Number of dissected Ap**	**Infection Rate **^**1**^	**Number of poolscreened Ap**	**PCR-Poolscreen Estimation **^**2**^
**Afareaitu**	216	8.3 [4.6–12]	660	2.5 [1.2–4.3]
**Haumi**	179	1.7 [0–3.6]	540	0.8 [0.2–2.0]
**Maatea**	478	1.3 [0.3–2.3]	1420	0.4 [0.2–1.0]
**Teavaro**	67	3 [0–7.1]	200	3.4 [1.0–8.0]
**Vaiare**	81	6.2 [1–11.4]	200	1.8 [0.3–5.1]

^1 ^infection rate [95% confidence interval]

^2 ^point estimate [95% confidence interval] (DNA extracted according the B method; sample fraction used 1/240^e^

**Figure 1:**
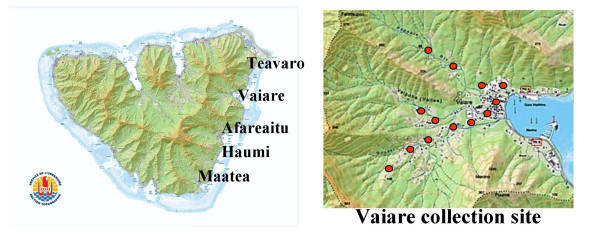
**Situation map of Moorea island (scale : 1 cm = 4 km)**. Left panel : Localization of the study area and the 5 collection sites Right panel : repartition of the collection "stations" in the collection site of Vaiare

**Figure 2:**
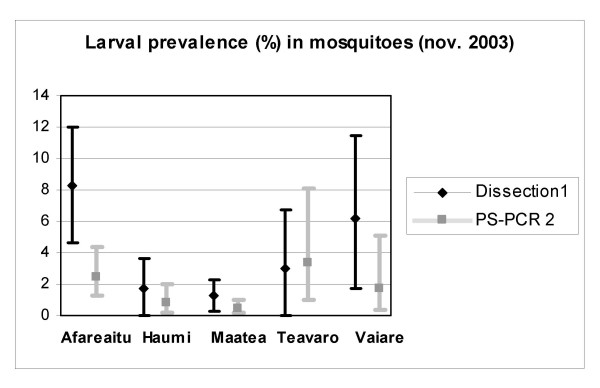
***W. bancrofti *(L1–L3) prevalence in dissected and PCR-poolscreened mosquitoes collected in november 2003**. ^1 ^prevalence [95% confidence interval] ^2 ^point estimate [95% confidence interval]

**Figure 3:**
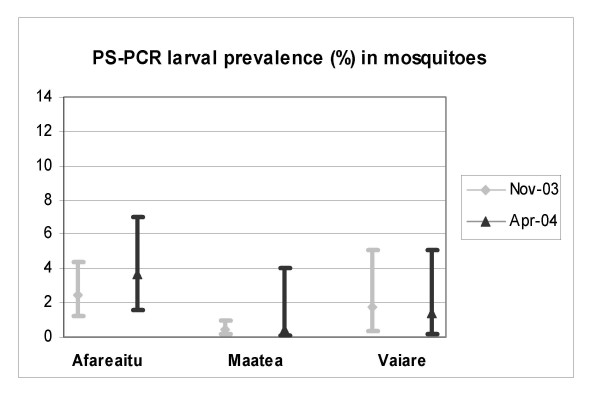
*W. bancrofti *(L1–L3) prevalence in PCR-poolscreened mosquitoes at 2 different seasonal periods.

**Table 6:** *Wuchereria bancrofti *larval (L1–L3) prevalence in PCR-poolscreened mosquitoes at 2 seasonal periods

**Collection** ** sites**	***November 2003*** ** Number of poolscreened Ap**	**PCR-Poolscreen** ** Estimation PCR**_**1**_	***April 2004 *** **Number of poolscreened Ap**	**PCR-Poolscreen** ** Estimation PCR**_**2**_
**Afareaitu**	660	2.5 [1.2–4.3]	340	3.7 [1.6–7.0]
**Maatea**	1420	0.4 [0.2–1.0]	480	0.4 [0.1–1.5]
**Vaiare**	200	1.8 [0.3–5.1]	160	1.4 [0.2–5.0]
**Total area**	**2280**	**1.1 [0.6–1.7]**	**980**	**1.5 [0.8–2.7]**

point estimate [95% confidence interval] (DNA extracted according the B method; sample fraction used 1/240^e^

## Discussion

Annual MDA with microfilarial drugs, the only currently available tool for combating filarial infection should be efficient, in principle, in eliminating lymphatic filariasis. Careful monitoring of filarial transmission is necessary to follow the progression towards elimination and particularly for deciding when to stop communitary treatment as well as to certify elimination.

Entomologic measures of filarial infection of vectors provide "real-time" estimates of filarial transmission [14]. Dissection is considered to be the gold standard against which other methods have to be compared. Procedures were designed to standardize our PCR method and to refine our protocol to assess filarial infection levels in mosquitoes. In a validation assay, we assessed filarial infection rates in field-captured *Ae. polynesiensis *from 5 areas located along the east-south-east coast of Moorea island in French Polynesia and compared PS-PCR with dissection.

Field-collections showed that mosquito population densities fluctuated significantly from one site to another. The sampling strategy aiming to collect samples representative of the mosquito populations in terms of density and infection status, appears to yield valuable information on the situation of the whole study area. Dissection work was performed by experienced entomologists with a special care (see method section) to avoid underestimation of infection status.

Infection rates determined by dissection (percentage of mosquitoes infected with any of the developing stages of *W. Bancrofti*) ranged from 1 to 8 % in the 5 sites sampled in November 2003 with a mean value of 3 (2-4) % for the total area. This value calculated from approximately one thousand dissected mosquitoes is a useful estimate of the "potential transmission index to humans" especially in the absence of recent data on the infection levels in the human population of the study area.

Repeated experiments to optimize a PS-PCR protocol using the Qiagen kit DNA extraction method (B method) were performed with the aim to yield results consistent with sensitivity obtained with the "reference" method (A method) for pools of up to 50 laboratory-reared mosquitoes spiked with experimentally infected material. The protocol was optimized for: (1) fine grinding of mosquitoes, (2) release of DNA by a double incubation with proteinase k, (3) washings of the DNA extract bound to the silica column to reduce inhibitors, (4) detection of false negatives by using an internal standard and (5) meticulous care to avoid false positives (contaminations) leading to successful positive and negative controlled experiments.

Validation of this PS-PCR protocol performed on mosquitoes collected in the study area, assaying 200 pools of 20 individuals, yielded estimated values of the larval prevalences in the area. Dissection and PS-PCR assay generated overlapping/similar although not identical results.

Although dissection is a very effective tool to monitor infection prevalences in vector populations and has been the gold standard, it is a very laborious, time-consuming method that requires experienced entomologists. In our situation, the decision to use PS-PCR rather than individual dissection even for prevalence values > 1% takes in account the scarcity of trained entomologists together with the fact that estimation of the filarial transmission can be conducted adequately on only a few hundreds of mosquitoes. When infection prevalence declines below 1%, only PS-PCR is cost effective. It was important to experiment a precisely calibrated and validated protocol before transmission level decrease.

Caution must be taken with this approach, however. First, *Ae polynesiensis *is a dengue as well as a filariasis vector and the absence of a commercially available trap to sample the mosquito population requires the use of human landing catches with the possibility of accidental biting. A second challenge is that in a MDA programme to eliminate lymphatic filariasis, the infection rates in the human and mosquito populations will diminish with successive treatments of the human population. Monitoring progress towards elimination will therefore require increasingly larger samples of mosquitoes to measure significant reductions in infections. Both of these obstacles could be overcome with the development of a trap capable to capturing large numbers of *Ae polynesiensis*.

In conclusion, the adapted PS-PCR technique able to detect one microfilaria in pools of up to 50 mosquitoes presented in this paper confirms that this assay is adequate for testing *Ae. polynesiensis *mosquitoes in the context of filariasis elimination programs. It could be usefully applied to monitor infection prevalence of *W. bancrofti *in the whole area where *Ae. polynesiensis *is the main vector when an efficient trap be developped.

## Competing interests

The author(s) declare that they have no competing interests.

## Authors' contributions

AML designed and coordinated the study, YS oversaw mosquito collections, dissections and rearing, CP conducted PCR assays, data entry and data analysis, and ND assisted with fieldwork design and statistical analysis.
