# Correction: PCR and dissection as tools to monitor filarial infection of *Aedes polynesiensis *mosquitoes in French Polynesia

**DOI:** 10.1186/1475-2883-6-5

**Published:** 2007-03-30

**Authors:** Catherine Plichart, Sandra J Laney, Yves Sechan, Neil Davies, Anne-Marie Legrand

**Affiliations:** 1 Institut Louis Malardé, PO Box 30, Papeete, 98713 Tahiti, French Polynesia; 2 Smith College, Clark Science Center, Northampton, MA 01063, USA; 3 Centre IRD (Institut de Recherche pour le Développement), PO Box 529, Papeete, 98713 Tahiti, French Polynesia; 4 Richard B. Gump South Pacific Research Station, University of California Berkeley, PO Box 244, 98728 Moorea, French Polynesia

## 

After publication of this work [1], we noted that we had inadvertently failed to include the complete list of all co-authors. The full list of authors has now been added and the Authors' contributions and Competing interests sections modified accordingly.

## Competing interests

The author(s) declare that they have no competing interests.

## Authors' contributions

AML designed and coordinated the study, YS oversaw mosquito collections, dissections and rearing, CP conceived and conducted PCR assays, data entry and data analysis, ND assisted with fieldwork design and statistical analysis, and SJL conceived of, and worked on the optimization of the PS-PCR assay.
